# Dual patch voltage clamp study of low membrane resistance astrocytes *in situ*

**DOI:** 10.1186/1756-6606-7-18

**Published:** 2014-03-17

**Authors:** Baofeng Ma, Guangjin Xu, Wei Wang, John J Enyeart, Min Zhou

**Affiliations:** 1 Department of Neuroscience, The Ohio State University Wexner Medical Center, Columbus, OH 43210, USA; 2 Department of Neurology, Tongji Hospital, Tongji Medical College, Huazhong University of Science and Technology, Wuhan 430030, P.R. China

**Keywords:** Astrocytes, Voltage clamp, Membrane resistance, Kir4.1, GABA_A_ receptor

## Abstract

Whole-cell patch clamp recording has been successfully used in identifying the voltage-dependent gating and conductance properties of ion channels in a variety of cells. However, this powerful technique is of limited value in studying low membrane resistance cells, such as astrocytes *in situ*, because of the inability to control or accurately measure the real amplitude of command voltages. To facilitate the study of ionic conductances of astrocytes, we have developed a dual patch recording method which permits membrane current and membrane potential to be simultaneously recorded from astrocytes in spite of their extraordinarily low membrane resistance. The utility of this technique is demonstrated by measuring the voltage-dependent activation of the inwardly rectifying K^+^ current abundantly expressed in astrocytes and multiple ionic events associated with astrocytic GABA_A_ receptor activation. This protocol can be performed routinely in the study of astrocytes. This method will be valuable for identifying and characterizing the individual ion channels that orchestrate the electrical activity of low membrane resistance cells.

## Introduction

When patch clamp was first applied to study glial cells in brain slices in the early 1990s, it became immediately clear that in a subpopulation of glial cells, characterized by a linear current-to-voltage (*I-V*) relationship passive K^+^ membrane conductance and low membrane resistance (*R*_M_), adequate voltage clamp quality could not be achieved [1,2]. In later studies, this subpopulation of glial cells has been unequivocally shown as the only electrophysiological phenotype of astrocytes in rat hippocampus when animals reach adulthood [3,4]. Therefore, the expression of passive membrane K^+^ conductance is a characteristic of functional mature astrocytes. In view of a widespread distribution of passive astrocytes in various brain regions and in species ranging from low amphibians to humans, it is possible that the distinctive passive K^+^ conductance and low *R*_M_ are common features of mature astrocytes in the brain.

Despite of an increasing awareness of the functional importance of astrocytes in the brain, the exceptionally low *R*_M_, estimated in the range of 2–5 MΩ, creates a formidable barrier for patch clamp voltage study of this major glial type. Specifically, the low *R*_M_ introduces a large error because a significant fraction of the voltage drop during a command potential occurs across the electrode tip rather than the cell membrane [5]. Because of this limitation, it has been difficult to identify and characterize the ion channels and transporter activities that establish the electrical properties of astrocytes. In particular, astrocytes are known to express a variety of K^+^ channels, including inwardly rectifying Kir4.1 and two-pore domain (K2P) TWIK-1 and TREK-1 leak-type K^+^ channels. The inability to effectively voltage clamp astrocytes has prevented these currents from being individually characterized with respect to their relative functional expression, conductance, rectification, contribution to *V*_M_, and modulation by neurotransmitters through activation of G-protein-coupled receptors. Therefore, an alternative electrophysiological method is urgently needed, and if such a new method could be successfully developed, voltage clamp study would be invaluable to gain insights into the basic electrophysiological properties of astrocytes in the adult brain.

### Rationale and validation of dual patch single cell recording technique

To circumvent the limitation imposed by conventional whole-cell patch clamp recording for study of low *R*_M_ astrocytes, we have developed a dual patch voltage clamp method. In this method, two patch electrodes are sealed to the cell body of a single astrocyte in the whole-cell configuration. This design has been conceived to overcome the following limitations inherited by single electrode voltage patch clamp for study of low *R*_M_ cells. In single electrode patch clamp recording, the *R*_M_ is typically calculated from the fit of a voltage command (*V*_C_) induced membrane current (*I*) near the resting membrane potential that carries a large error in low membrane resistance cells (see details in the following sections). In contrast, in dual patch recording, the *V*_C_ is applied through one patch electrode, while a second electrode is used to measure the *V*_C_-induced actual change in membrane potential (*V*_M_). Using this method, an accurate current-to-voltage (*I-V*_M_) relationship can be obtained over a wide range of potentials, and the voltage-dependent membrane conductance can be reliably calculated. An additional unique opportunity offered by dual patch recording is that the *I*, *V*_M_, and *R*_M_ can be measured and calculated simultaneously. This, however, needs to be experimentally validated.

In the present study, we started with a direct measurement of voltage errors in low *R*_M_ astrocytes with dual patch recording. That was followed by the validation of dual patch recording for reliable and accurate measurement of *R*_M_. Building upon these, we further showed that the voltage dependent activation kinetics of inwardly rectifying K^+^ channel conductance can be established and pharmacologically studied. Finally, astrocytic ionotropic GABA_A_ receptor was used as an example to show the simultaneous measurement of *I*, *V*_M_, and *R*_M_ and disclosure of multiple ionic events associated with astrocytic GABA_A_ receptor activation.

#### A lack of voltage control in astrocyte recording in situ

**Figure 1:**
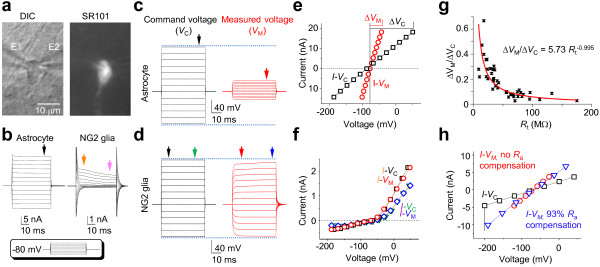
**Poor voltage clamp quality in astrocyte recording. (a)** DIC and SR101 fluorescent images of a dual patch recorded astrocyte in the hippocampal CA1 *stratum radiatum* region; electrode 1 (E1) and electrode 2 (E2) were in voltage clamp and current clamp modes, respectively. **(b)** Whole-cell current profiles of an astrocyte and an NG2 glia are shown as indicated; the cells were held at resting (-80 mV), then stepped to command voltages from -180 mV to +20 mV with 20 mV increments (inset). **(c, d)** Comparison of command voltages (*V*_C_) applied through E1 and the resultant potentials across the membrane (*V*_M_) measured from E2 for the recorded astrocyte and NG2 glia, respectively. **(e, f)** The *I*-*V*_C_ and *I*-*V*_M_ were plotted in the same chart for voltage clamp quality comparison. **(g)** The *R*_t_ was plotted against *V*_M_/Δ*V*_C_ (*n* = 44) and fitted with Δ*V*_M_/Δ*V*_C_ = *aR*_t_^*b*^; the *V*_M_/Δ*V*_C_ decreases with increasing *R*_t_, which yields *R*_M_ and exponent values of 5.73 MΩ and -0.995, respectively. **(h)** Comparison of *I*-*V*_M_ relationships in the presence and absence of *R*_a_ compensation, showing a good voltage clamp quality after *R*_a_ compensation.

Astrocytes were identified for recording based on cell soma morphology and SR101 staining in the CA1 *stratum radiatum* (Figure 1a). Consistent with our previous report, functional mature hippocampal astrocytes after postnatal day 21 identically express a linear current-to-voltage (*I-V*) relationship membrane K^+^ conductance, or passive K^+^ conductance (Figure 1b) [3]. To directly assess the voltage clamp quality, a MultiClamp 700B amplifier designed for dual channel recording was used. Two patch electrodes were sealed sequentially on a single astrocyte. Electrode one (E1) and two (E2) were maintained in voltage clamp and current clamp mode, respectively (Figure 1a). The same recording configuration was also used for NG2 glia for comparison of voltage clamp quality. NG2 glia are morphologically similar to astrocytes in terms of the soma shape *in situ*, but can be identified based on the absence of SR101 staining and expression of voltage-gated K^+^ channel conductances. In both cases, the *V*_C_ was applied through E1, which induced a characteristic passive K^+^ conductance in the low *R*_M_ astrocyte, and outward voltage-gated transient and delayed rectifier K^+^ channel currents in the high *R*_M_ NG2 glia (Figure 1b). In E2, the *V*_C_-induced actual *V*_M_ was recorded simultaneously (Figure 1c, d). Both *I*-*V*_C_ and *I-V*_M_ were plotted for comparison of voltage clamp quality (Figure 1e, f), where the *V*_M_ was only 19.0 ± 1.7% of the *V*_C_ in astrocytes (*n* = 54), in contrast, *I-V* curves in NG2 glia were nearly superimposable. The plots shown in Figure 1e, f further display a striking difference in the voltage clamp quality between these two glial subtypes.

Since the total membrane resistance (*R*_t_) is the sum of access resistance (*R*_a_) and membrane resistance (*R*_M_) in series [6], to confirm that the dual patch results obey the basic voltage-division principle as in single electrode patch clamp recording, we used Δ*V*_M_/Δ*V*_C_ as an indicator of voltage clamp quality that theoretically follows a relationship of Δ*V*_M_/Δ*V*_C_ = *R*_M_*R*_t_^-1^. We plotted *R*_t_ against Δ*V*_M_/Δ*V*_C_ and fit the data with Δ*V*_M_/Δ*V*_C_ = *aR*_t_^*b*^ from 44 dual patch recordings (Figure 1g). Of note, to obtain an accurate fit, the *R*_t_ greater than 30 MΩ that are routinely discarded in study were included in this analysis. As shown in the Figure 1g, the fit yielded a *R*_M_ (*a*) of 5.73 MΩ that was almost identical to the measured *R*_M_ (see next section). Importantly, the fit yielded an exponent (*b*) of -0.995, which matches closely to a theoretically ideal value of -1, indicating that the results derived from the two electrodes in dual patch recording follow accurately to the voltage-division principle.

In dual patch recording, E1 and E2 are separated by 3.4 ± 0.2 μm (n = 15), and the *V*_M_ measured from E2 (*V*_M,2_) is used to represent the *V*_M_ in E1(*V*_M,1_) to establish the actual *I*-*V*_M_ relationship, therefore to what extent does *V*_M,2_ reflect the intended *V*_M,1_ is an issue to be resolved for the concern of space clamp error, especially for low *R*_M_ astrocytes. To estimate *V*_M,1_ in E1, we assumed that *R*_a_ is 1.5-fold of the recording electrode resistance (*R*_p_) and used a -40 mV *V*c to induce current (*I*). Subsequently, the *V*_M,1_ is calculated from (*V*c-1.5 *R*_p_*I)/*I*, and the fidelity of *V*_M,2_, in the form of *V*_M,2_/*V*_M,1_, is estimated at 93.9% (n = 4). Thus, within a tip-to-tip distance < than 4 μm in our study, *V*_M,2_ can be used with high fidelity for *I*-*V*_M_ plot and for the *R*_M_ measurement that will be described in the following section.

**Figure 2:**
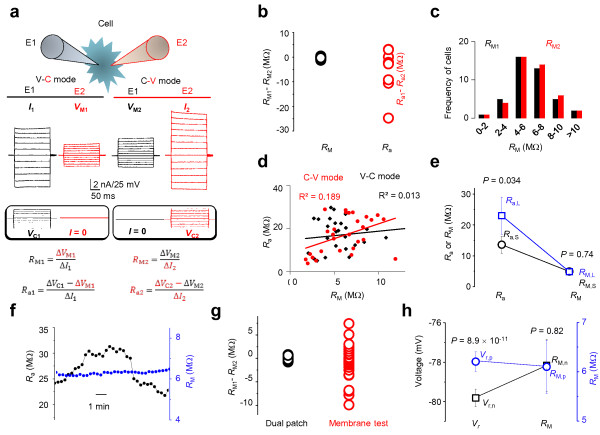
**Accurate *R***_**M **_**measurement in dual patch recording. (a)** Voltage (*V*) and current (*C*) modes were alternated between E1 and E2 to obtain two sets of *R*_M_ and *R*_a_ values for comparison. **(b)** Open black and red circles are *R*_M1_-*R*_M2_ and *R*_a1_-*R*_a2_ values, respectively (*n* = 44). Despite a large variation in *R*_a1_-*R*_a2_, the *R*_M1_-*R*_M2_ varies minimally in the same cells. **(c)** The *R*_M1_ (black, from E1) and *R*_M2_ (red, from E2) values were sorted into 2 MΩ intervals and plotted against their respective occurrence frequencies; the frequency distribution patterns are nearly identical for the data obtained from E1 and E2 (*n* = 54). **(d)** The pooled *R*_a_ and *R*_M_ values are not correlated. **(e)** Variation of *R*_a_ between the two electrodes does not affect the accuracy of *R*_M_ measurement. **(f)** The long-term *R*_M_ recording remains stable and is independent of variation in *R*_a_. **(g)***R*_M1_-*R*_M2_ varies minimally in dual patch recording compared to measurements obtained from the “membrane test” (pClamp 9.0 software). **(h)** Within the acceptable range of resting potentials (*V*_r_), *V*_M_ does not affect the accuracy of *R*_M_ measurement.

NG2 glia typically exhibits a *R*_M_ greater than 200 MΩ [7], or a *R*_a_/*R*_t_ ratio of ~1/20; therefore, greater than 95% of the *V*_C_ is distributed across *R*_M_, permitting good voltage clamp study (Figure 1d) as compared to the poor clamp quality in astrocytes. To determine the extent to which *R*_a_ compensation could be useful for improving the voltage clamp quality in low *R*_M_ astrocyte, we first used E2 as a voltage monitor in dual patch recording to guide the adjustment of *R*_a_, C_M_ and prediction/correction parameters in E1. Occasionally, it was possible to achieve a nearly perfect voltage control (Figure 1h). However, without E2 as a voltage referee, conventional empiric guided R_a_ compensation resulted in a largely variable range of voltages, specifically, the voltage improvement varied from 40% to 143% from 27 trials in 3 cells. Additionally, the *R*_a_ typically varies significantly during the recording (see Figure 2f), thus, it was impossible to use a preset *R*_a_ compensation parameters in the entire course of recording. Therefore, *R*_a_ compensation is not practically useful for clamp quality improvement in low *R*_M_ astrocyte.

#### R_M_ can be accurately measured in dual patch single cell recording

The low *R*_M_ of passive astrocytes is the cause of poor voltage clamp quality. In dual patch recording, the *V*_C_-induced *I* and the actual voltage drop across the membrane *V*_M_, are measured separately by the two electrodes, so that *R*_M_ can be calculated accurately from *R*_M_ = *V*_M_/*I*. Using dual patch recording, the voltage and current clamp modes can be alternated between E1 and E2 (Figure 2a), allowing *R*_M_ and *R*_a_ to be measured for each electrode from the same cell. We therefore analyzed these results to determine 1) whether *R*_M1_ and *R*_M2_ are identical in the same cell, as expected, and 2) whether the *R*_M_ in dual patch recording is truly independent of the variation of *R*_a_. We used the difference in measured access resistances (*R*_a1_-*R*_a2_) and membrane resistances (*R*_M1_-*R*_M2_) to determine the variation of these two parameters in the same cell. The results showed that, in the same cell, the *R*_a_ varied considerably between E1 and E2 (Figure 2b, red open circles), while the *R*_M1_ and *R*_M2_ were essentially identical (Figure 2b, black open circles). We next compared the measured *R*_M_ values from E1 and E2 and found that these were nearly identical (*R*_M1_, 6.00 ± 0.32 MΩ *vs. R*_M2_, 6.12 ± 0.33 MΩ, *n* = 54, *P* = 0.79). Finally, we sorted *R*_M1_ and *R*_M2_ data sets separately with 2 MΩ intervals and plotted them against their respective occurrence frequencies to determine the pattern of frequency distributions. As shown in Figure 2c, the frequency distribution of the data sets between *R*_M1_s and *R*_M2_s matched almost perfectly (*n* = 54), which is consistent with the view that *R*_M_ can be reliably measured in dual patch recording.

Theoretically, *R*_M_ measurement is independent of *R*_a_ variation in dual patch recording. This was confirmed from the following experiments. First, in the correlation analysis shown in Figure 2d, *R*_M_ is not correlated with *R*_a_. Second, the *R*_a1_ and *R*_a2_ measured from the same cell typically differ from each other, we next sorted out the large *R*_a_ (*R*_a,L_) and small *R*_a_ (*R*_a,S_) data sets into two groups from 10 dual patch recordings (Figure 2e). As anticipated, the *R*_a,L_ and *R*_a,S_ varied significantly (*R*_a,L_, 23.02 ± 6.07 MΩ *vs. R*_a,S_, 13.6 ± 2.8 MΩ, *n* = 10, *P* = 0.034), but their corresponding *R*_M,L_ (4.85 ± 0.74 MΩ) and *R*_M,S_ (4.90 ± 0.77 MΩ) were almost identical (*n* = 10, *P* = 0.74). Third, while the *R*_a_ varies considerably during long-term recording, the simultaneously measured *R*_M_ remained constant (Figure 2f).

In pClamp software, *R*_M_ can be measured from the “Membrane test” program, which has been widely used for study of astrocyte electrophysiology [5,8]. We found a much large variation between *R*_M1_ and *R*_M2_ data sets obtained from “Membrane test” than the data set from dual patch recording (Figure 2g), indicating that dual patch offers a more reliable and precise *R*_M_ measurement.

It should be noted that in single electrode recording, *R*_a_ is resolved from time constant (τ), i.e., τ= *R*_a_*C*_M_. The accuracy of *R*_a_ estimation depends on a close approximation of *R*_a_ to the effective access resistance (*R*_a,eff_); that is *R*_a,eff_ = *R*_a_*R*_M_/(*R*_a_ + *R*_M_) [9]. In high *R*_M_ cells, such as neurons, *R*_M_ > > *R*_a_, so that *R*_a,eff_ ≅ *R*_a,_ and *R*_a_ can be resolved with good confidence. However, in low *R*_M_ astrocytes, because *R*_a,eff_ ≅ *R*_M_, the *R*_a_ measured from “Membrane test” (pClamp9.0) is erroneous, so is *R*_M_. The latter is calculated from *R*_M_ = *R*_total_-*R*_a_. Although we know that *R*_M_ in astrocyte is extremely low, but reliable *R*_M_ measurement is not yet available for the noted reason. Accordingly, our report provides a yet most reliable *R*_M_ measurement from mature astrocytes.

Within the acceptable whole-cell resting membrane potentials (*V*_r_), i.e., more negative than -75 mV [10], the *V*_r_ values always vary slightly between E1 and E2 in the same cell. To ensure that within the accepted *V*_r_ range, variation of *V*_r_ does not affect *R*_M_ measurement, we sorted all the positive *V*_r_ (*V*_r,p_) and negative *V*_r_ (*V*_r,n_) from 19 dual patch recordings into two separate groups for *R*_M_ comparison. While the *V*_r,p_ (-77.97 ± 0.50 mV) and the *V*_r,n_ (-79.81 ± 0.45 mV) varied significantly (*n* = 19, *P* = 8.91 × 10^-11^), the *R*_M_ was essentially the same between the two groups (*R*_M,p_, 6.10 ± 0.54 MΩ *vs. R*_M,n_, 6.13 ± 0.53 MΩ, *n* = 19, *P* = 0.83) (Figure 2h).

The experiments described above used young adult mice of 3–4 weeks old. To determine if this protocol is also applicable for astrocytes from fully mature mice brain, we next prepared slices from 5-month old mice. We found that dual patch single astrocyte recording remains feasible. At this animal age, dual patch recording revealed a similar *V*_r_ of -78.75 ± 0.47 mV (*n* = 7), and a *R*_M_ of 5.91 ± 1.52 MΩ (*n* = 5) compared to young adult mice.

In summary, we show that in dual patch recording, the *R*_M_ can be measured accurately and reliably, and the majority of astrocytes exhibit *R*_M_ values in the range of 4–8 MΩ. Importantly, since astrocytes are non-excitable, most of their ionic events can be monitored directly by *R*_M_ measurement. Thus, the ability of dual patch to permit long-term stable *R*_M_ measurement should be highly useful for pharmacological analysis of functional proteins in astrocytes, such as K^+^ channels and transporters.

#### The temporal resolution in astrocyte dual patch recording

**Figure 3:**
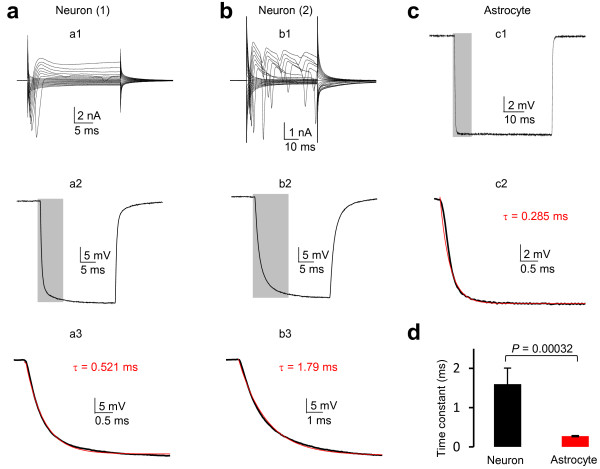
**A rapid time constant in dual patch single astrocyte recording. (a, b)** Whole-cell current profiles of two interneurons recorded from CA1 *stratum radiatum*. (a1, b1) To resolve the time constant (τ) of *V*_C_ induced *V*_M_ in dual patch recording, a -40 mV voltage pulse was applied through E1 and the induced *V*_M_ was recorded from E2. (a2, b2) The downward rise phase of *V*_M_s (from the grey shadows in a1, b1) were fitted with mono-exponential model to resolve the τ values, which varied considerably between a2 and b2. **(c)** A representative -40 mV voltage pulse induced *V*_M_ from dual patch astrocyte recording (c1) and the resolved τ value (c2). **(d)** Summary of the τ values from interneurons and astrocytes, indicating a significantly faster τ value in astrocyte compared to that of the neurons in dual patch recording.

We next sought to answer how a compromised *V*_C_ affects the temporal resolution of the measured *V*_M_ from the E2, as poor temporal resolution hampers the ability of detecting ion channels with fast activation/inactivation kinetics, To address this, we dual patch recorded interneurons in CA1 *stratum radiatum* as control group for comparison of temporal resolution. Consistent with a previous report that hippocampal interneurons are morphologically and functionally diverse [8], the whole-cell current profile varied from cell to cell (Figure 3a, b). Nevertheless, interneurons do not express hyperpolarization-induced inward currents and showed a high *R*_M_ of 155 ± 41 ΩM (*n* = 4). Therefore, we applied a -40 mV voltage pulse through E1 to induce *V*_M_ change in E2. The time constant (τ) of *V*_M_ onset was used to analyze and compare the temporal resolution between neurons and astrocytes (Figure 3, a2, b2, c1), and τ was resolved from mono-exponential fit of the downward rise phase of *V*_M_ (Figure 3, a3, b3, c2). The τ varied from cell to cell in neurons with an average value of 1.60 ± 0.41 ms (*n* = 4). The τ varied much less among astrocytes (Figure 3c), 0.277 ± 0.015 ms (*n* = 9), which is significantly faster than that of interneurons.

Theoretically, a higher temporal resolution in astrocytes should also be attributable to the low *R*_M_. Because τ is the product of effective access resistance (*R*_a,eff_) times *C*_M_, where *R*_a,eff_ = *R*_a_.*R*_M_/(*R*_a_ + *R*_M_) [9]. As noted above, the *R*_a,eff_ approximates to *R*_M_ in low *R*_M_ astrocytes, which results in a faster τ compared to high *R*_M_ neurons.

Because of an extremely high leak K^+^ conductance, it is worth noting that the likelihood to observe a voltage dependent conductance in astrocyte whole-cell current depends chiefly on the expression quantity of the candidate channel, but not the temporal resolution of dual patch recording.

### Experimental design

The following issues should be considered in the experimental design.

**Figure 4:**
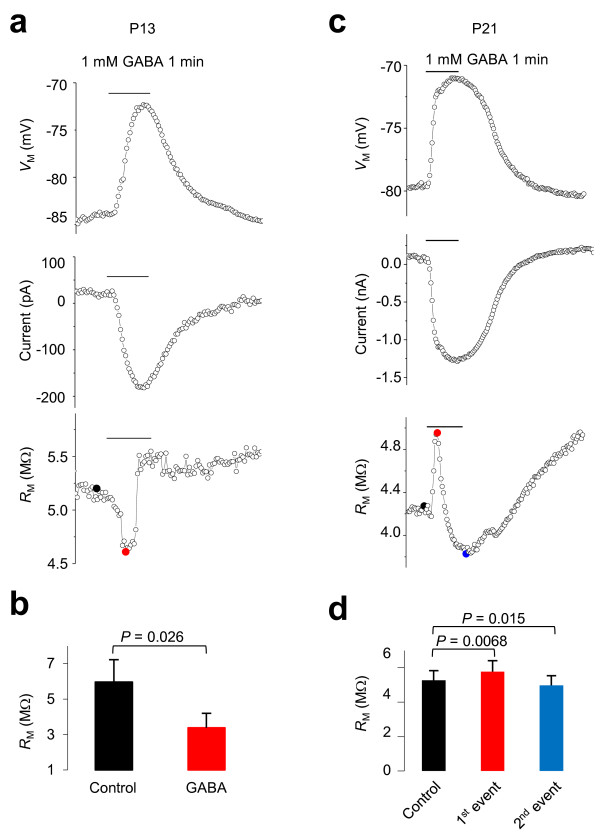
**Disclosure of inwardly rectifying K**^**+ **^**conductance in dual patch recording. (a, b)** Bath application of 100 μM Ba^2+^ produce a moderate *V*_M_ inhibition but not noticeable inhibition of inward currents using the conventional single electrode recording, although the *V*_M_ depolarizes by 2.6 mV with full recovery. Whole cell currents were induced by voltage steps from -210 to 30 mV with 30 mV increments. A pre-pulse voltage at 0 mV/300 ms was used to maximally activate Kir4.1 currents (inset). **(c)** Application of 100 μM BaCl_2_ in dual patch induces a significant increase in *R*_M_. **(d)** An example shows *I*-*V*_M_ relationships before (black) and after (red) 100 μM Ba^2+^ application. Ba^2+^ sensitive currents were resolved by subtraction of currents in the presence of Ba^2+^ application from the control. **(e)** A negative *V*_M_-dependent *G*_M_ increase is inhibited totally by 100 μM Ba^2+^. Voltage dependent Ba^2+^ sensitive conductances are shown in **(f)**.

(1) The advantage of using a combination of MultiClamp 700B (or 700A) amplifier and pClamp 9 (or 10) software is that the voltage and current clamp mode can be designated either the same or differently between E1 and E2. As the illustration shown in Figure 2a, E1 and E2 can be initially programed in voltage (V) and current (C) clamp mode, respectively, and then swapped in the following experiment. For the electrode in V mode, the controlled input is *V*_*C*_ and the recorded signal is *I*. For the electrode in C mode with no holding current, the recorded signal is the *V*_M_ that is induced by *V*_C_ from another electrode in the pair. The voltage dependence and activation kinetics of ion channels of interest can then be constructed by plotting the measured *I* values against their corresponding *V*_M_ values (Figure 2a).

(2) The measured *V*_M_ from E2 can be much smaller than that of the applied *V*_C_ in E1, which could make accurate *V*_M_ calculation difficult. When *V*_M_ cannot be detected with confidence, increase *V*_C_ to ensure a reliable *V*_M_ reading. Likewise, it is beneficial to set up one of the electrodes in the pair with a relatively small *R*_t_ in V mode.

(3) The high leak K^+^ conductance is the cause of difficulty for voltage clamp identification and pharmacological study of a specific K^+^ channel of interest. The following examples will demonstrate how a combined pharmacology and current subtraction strategy can be used to overcome this obstacle in dual patch recording.

(4) Channel inhibitors or activators are not yet available for most of two-pore channels, use of genetically modified mice, such as TWIK-1 and TREK-1 knockout mice, should be highly valuable to gain insights into the molecular identify and function of astrocyte passive conductance in the future [10].

(5) The procedure described here should be equally useful for study of other cell type with similarly low *R*_M_. When identification of ion channel with fast activation/inactivation kinetics is the purpose of study, a low *R*_a_ in both electrodes is essential and the commonly used conventions for single electrode recording also apply to dual patch recording.

(6) When set up E1 and E2 in V and C mode, respectively, E2 only observes the deviation of *V*_M_ from the intended *V*_C_. The measured *V*_M_ from E2 is not compensated for either dynamic or steady-state voltage errors. The former error is associated with the onset of a *V*_C_ and is proportional to the time constant (τ=*R*_a_*C*_M_). The latter refers to the deviation of *V*_M_ from the steady-state holding *V*_C_ in response to rapidly activated ionic conductances, such as ionotropic GABA_A_ receptor in astrocytes (Figure 4). As noted above, effective *R*_a_ compensation cannot be reliably performed without the guidance of E2 as voltage monitor. Also, astrocytes show a faster *V*_M_ onset temporal resolution and unlikely express large amount of ion channels with rapid activation kinetics. Therefore, unless an experiment is designed to target on a specific voltage gated ionic conductance, *R*_a_ compensation is not recommended.

**Figure 5:**
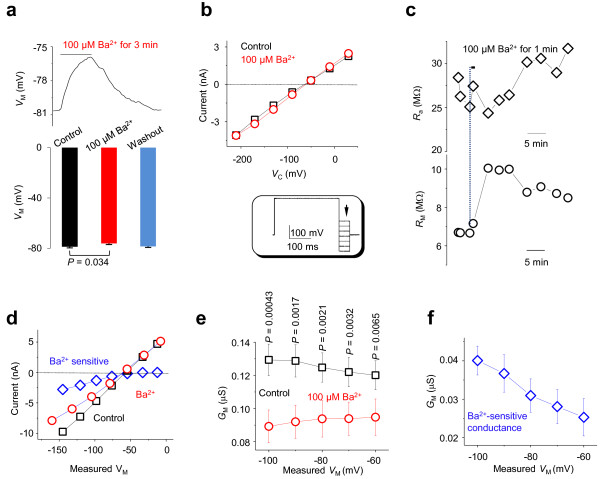
**Simultaneous acquisition of *V***_**M**_**, *I***_**M**_**, and *R***_**M **_**in dual patch recording. (a, b)** Bath application of 1 mM GABA induced *V*_M_ depolarization, and inward currents were measured simultaneously by the two electrodes. The measurements are identical in astrocytes from P13 **(a)** and P21 **(c)** mice. However, only in the *R*_M_ measurement of P21 astrocytes, three ionic events are temporally distinct **(c)**. **(b)** Quantification of a GABA-induced *R*_M_ decrease in P9-P13 mice. **(d)** Quantification of a GABA-induced *R*_M_ change in P21 mice. The control, first, and second events correspond to the black, red and blue filled circles shown in the bottom panel of **c,** respectively.

(7) Other electrophysiological methods, such as discontinuous single electrode voltage clamp (dSEVC) and two electrode voltage clamp (TEVC) technique, typically use sharp electrode, which is technically unfeasible for mammalian astrocytes with a some diameter less than 10 μm. Additionally, no validated and commercially available amplifiers are yet available for the intended goal of present research. Therefore, at least in the current stage, these techniques cannot be considered as alternatives.

### Advantages

#### Measurement of voltage-dependent membrane conductance: inwardly rectifying K^+^ channels

Since *V*_M_ can be accurately measured in E2 when command voltages are applied through E1, the *I-V* relationship can be accurately established in low *R*_M_ astrocytes. To demonstrate this experimentally, we focused on the inwardly rectifying Kir4.1 K^+^ channel, which expresses abundantly in astrocytes [11]. It has been shown in cultured astrocytes that 100 μM Ba^2+^ inhibits Kir4.1 currents fully and depolarizes *V*_M_ significantly [12]. In single electrode recording from astrocytes in slices, although 100 μM Ba^2+^ also depolarized membrane potential by 2.6 mV (-78.7 ± 1.1 mV in aCSF *vs*. -76.1 ± 1.0 mV in BaCl_2_, *P* = 0.034, *n* = 7, Figure 4a), the degree of *V*_M_ depolarization is much less than cultured astrocytes, and the overall passive conductance was not noticeably altered. The difficulty in resolving Kir4.1 currents with single electrode voltage clamp recording is shown in Figure 4b. To maximally activate Kir4.1 currents, a 300 ms pre-pulse to 0 mV was used prior to the stepwise command voltages (inset, Figure 4b) [12]. These yielded no indication of inward rectification of whole-cell currents. In addition to a complex expression of other K^+^ channels that obscure the activation kinetics of Kir4.1 in astrocytes [11], poor voltage clamp quality be majorly is likely responsible for this.

In dual patch *R*_M_ measurement, one minute 100 μM Ba^2+^ bath application increased *R*_M_ from the control 7.46 ± 1.2 MΩ to 13.7 ± 3.1 MΩ (*n* = 7), corresponding to a membrane conductance (*G*_M_) decrease from 0.134 μS in the control to 0.073 μS in the Ba^2+^. Based on this analysis, Kir4.1 accounts for ~45.5% of the *G*_M_ (Figure 4c).

As the *V*_C_-induced actual *V*_M_ can be reliably determined to construct the actual *I-V*_M_ relationship for a conductance of interest, we next adjusted the *V*_C_ to reach the intended *V*_M_ from -60 mV to -100 mV, where the inward rectification kinetics of Kir4.1 should be evident [12]. The *I*-*V*_M_ curves shown in Figure 4d revealed a selective inhibition of inward whole-cell currents by Ba^2+^. To analyze the results statistically, we used voltage dependent *G*_M_ to describe the rectification characteristics. We show that a voltage-dependent inward *G*_M_ was clearly evident in the control condition (Figure 4e-f), which is consistent with the presence of functional Kir4.1 currents. Furthermore, this inwardly rectifying conductance was totally inhibited by 100 μM Ba^2+^. These results demonstrate that dual patch allows reliable establishment of *I-V*_M_ relationship and, in the present case, enables the functional identification and pharmacological analysis of inwardly rectifying Kir4.1 channels in astrocytes.

#### Identification of multiple ionic events associated with GABA_A_ receptor activation

A unique technical advantage of dual patch recording is that *I*, *V*_M_ and *R*_M_ can be measured and resolved simultaneously. We chose the astrocytic GABA_A_ receptor as an example to experimentally demonstrate the feasibility of this powerful application.

We have recently shown that, while GABA_A_ activation decreases *R*_t_ in both neurons and NG2 glia, GABA_A_ activation increases *R*_t_ in astrocytes, which is likely caused by a rapid secondary K^+^ channel inhibition [13]. However, single electrode recording in either voltage or current clamp mode does not provide a temporal resolution to separate these ionic events in astrocytes. Therefore, we further explored this issue in dual patch recording, where GABA-induced changes in *V*_M_, *I* and *R*_M_ can be obtained simultaneously. Interestingly, astrocytes at different developmental stages responded to GABA differently. At P13, immature astrocytes responded to 1 mM GABA application by *V*_M_ depolarization, activation of inward currents and *R*_M_ decrease (5.96 ± 1.26 MΩ in control *vs*. 3.37 ± 0.82 MΩ in GABA, *n* = 5, *P* = 0.026) (Figure 5a-b), responses which were identical to neuronal GABA_A_ receptor activation [13]. By comparison, in mature P21 astrocytes, while 1 mM GABA also induced inward currents and *V*_M_ depolarization, three obvious ionic events could be resolved in *R*_M_ measurements (Figure 5c-d). Specifically, the *R*_M_ first increased from the control 5.27 ± 0.56 MΩ to 5.77 ± 0.63 MΩ (*n* = 6, *P* = 0.0068), then decreased significantly (4.97 ± 0.57 MΩ, *n* = 6*, P* = 0.015). This effect was followed by a second sustained *R*_M_ increase, implying a complex modulation of astrocyte membrane conductance upon GABA_A_ activation.

As noted above, simultaneous measurement of *I*, *V*_M_ and *R*_M_ is feasible in dual patch recording. Additionally, only in this recording mode, multiple ionic events associated with astrocytic ionotropic GABA_A_ receptor activation could be readily identified. This powerful application should also be applicable for revealing complex ionic events in other cell types, such as neurons.

#### Summary of technical advantage of dual patch single astrocyte recording

1. As noted before, the *R*_a_ and *R*_M_ measurement from single electrode “Membrane test” software program are erroneous; this protocol allows accurate, stable and long-term *R*_M_ measurement from astrocytes. For non-excitable cells, this should have a broad usage for reliable pharmacological analysis of ion channel and electrogenic transporter activity in astrocytes.

2. An important purpose of this protocol is to validate whether the voltage dependence of a specific ionic conductance can be established and analyzed. We show from the *G*_M_ analysis of Kir4.1 currents that this is feasible (Figure 4).

3. A unique advantage we demonstrated in this protocol is that membrane *I*, *V*_M_, and *R*_M_ can be measured simultaneously in dual patch recording model. As shown in the example of astrocytic GABA_A_ receptor study, multiple ionic events can be disclosed following to GABA_A_ receptor activation (Figure 5).

4. Although it may not be highly useful, using E2 as a monitor, it is possible to achieve a nearly perfect voltage clamp quality with *R*_a_ compensation function (Figure 1h). This can not be done in single electrode voltage clamp recording.

### Potential limitation

The voltage clamp method is designed to reveal the voltage dependence of membrane conductances. Using Kir4.1 channel currents as an example (Figure 4), we demonstrated that the voltage dependence can be accurately established and pharmacologically analyzed in dual patch recording. However, even though the temporal resolution of *V*_M_ onset is faster in astrocytes (Figure 3), identification of low density expressing voltage-dependent ion channel conductance in low *R*_M_ astrocytes remains challenging. For example, immature astrocytes express voltage- and time-dependent outward transient K^+^ channels [3], but whether the expression of these channels continues in mature astrocytes cannot be answered with confidence with dual patch recording. Nevertheless, there is no evidence indicating extensive expression of voltage gated ion channels in astrocytes [11].

It is worth noting that two recent reports have successfully used dual patch single astrocyte recording to correct voltage error for analyzing the relative glutamate vs. Cl^-^ permeability ratio through Best1 channel [14], and identifying pH sensitive and anesthetic-sensitive leak K conductances in hippocampal astrocytes [15]. Thus, the dual patch method validated in this study provides a powerful means for studying the voltage dependence of leak type K^+^ channels, slow cycling transporters, and pumps that are abundantly expressed in astrocytes.

## Materials

### Reagents

• CaCl_2_, glucose, HEPES, KCl, KOH, Mg-ATP, MgCl_2_, Na_2_-GTP, NaCl, NaHCO_3_, NaH_2_PO_4_ (all from Sigma-Aldrich, St. Louis, MO)

• Sulforhodamine 101 (SR101) from Invitrogen (New York, NY)

• Deionized water with 18.2 MΩ•cm at 25°C ▲CRITICAL The quality of water is especially important for internal solutions.

• C57BL/6 J mice from postnatal day (P) 9–30, and 5 months old were used in different experiments noted. ! CAUTION All animal studies must be approved by the Institutional Animal Care and Use Committee. All the relevant ethics regulations have to be strictly followed. ▲CRITICAL The animal age needs to be carefully chosen depending on experimental purpose and we chose to use animals older than postnatal day 21 in most of the experiments as hippocampal astrocytes become mature after this postnatal day. To validate this protocol for study astrocytes from fully mature mice, 5-month old mice were used in some of experiments.

### Equipment

• An electrophysiology setup for submerged slices equipped with ×4 and ×40 infrared differential interference contrast (IR-DIC) visualization, a patch-clamp amplifier capable of current and voltage clamp and providing two channel recording (e.g., MultiClamp 700A or 700B, Molecular Devices), an interface for converting digital-analog signals between the amplifier and a computer (e.g., Digidata 1322A, Molecular Devices).

• (Optional) A fluorescent imaging system (e.g., Polychrome V system from Till Photonics, Germany) is advantageous for high resolution visualization of small glial soma and placing of dual patch electrodes on it. This system can also be used for identifying astrocyte based on SR101 positive staining. This system can also be used for simultaneous dual patch and ion sensitive dye measurement.

• Two micromanipulators for automatic loading of two electrodes to the top of the brain slice can shorten the loading time (e.g., PatchStar from Scientifica, UK).

• Commercially available borosilicate glass capillaries, such as the one from Warner Instruments (Cat. no. 64–0772), worked well in this experiment. An open electrode resistance in the range of 2.5-5 MΩ, when filled with KCl-based electrode solution, is well suited for dual patch recordings. However, for low *R*_M_ astrocytes, electrode resistance in the low-end of this range is recommended.

## Reagent setup

**Brain slice cutting solution** (in mM): 125 NaCl, 3.5 KCl, 25 NaHCO_3_, 1.25 NaH_2_PO_4_, 0.1 CaCl_2_, 3 MgCl_2_, and 10 glucose. Cutting solution should be freshly prepared on the day of the experiment.

**Artificial cerebral spinal fluid (aCSF)** (in mM): 125 NaCl, 3.5 KCl, 25 NaHCO_3_, 1.25 NaH_2_PO_4_, 2 CaCl_2_, 1 MgCl_2_, and 10 glucose (osmolality, 295 ± 5 mOsm) at room temperature (20–22°C).

**Electrode solution** (in mM): 140 KCl, 0.5 CaCl_2_, 1 MgCl_2_, 5 EDTA, 10 HEPES, 3 Mg^2+^-ATP, and 0.3 2Na^+^-GTP. This solution was titrated with KOH to pH 7.25-7.27, and the final osmolality was 280.0 ± 5.0 mOsm. The solution can be prepared in advance and stored at -80°C in ~0.5 ml aliquots.

**SR101:** Stock solution, 6 mM; working solution, 0.6 μM.

## Procedure

**1 |** Use a commercially available Vibratome, e.g., Pelco 1500, to prepare 250 μm thickness coronal hippocampal slices from mouse brain with a standard procedure we and others described before [5,16]. All solutions should be continuously bubbled with 95%O_2_/5%CO_2_.

**2 |** Place acute slices in a nylon net basket slice holder immersed in aCSF chamber for recovery and storage. The aCSF is continuously bubbled with 95%O_2_/5%CO_2_ for a typical experiment day of 6 to 8 hrs.

**3 |** For SR101 staining, transfer slice containing basket to another aCSF chamber containing 0.6 μM SR101 at 34°C for 30 min. Then, transfer the same basket back to the normal aCSF at room temperature for at least 1 h before experiment.

**4 |** For recording, transfer one brain slice to the recording chamber, lay it down on the bottom of glass chamber and gently place a platinum slice anchor (Warner Instruments) on the top of slice to prevent movement of slice from bath perfusion. Perfuse oxygenated aCSF at a rate of 2.5 ml/min.

**5 |** Fill two patch electrodes with electrode solution and install them to the electrode holders. Apply a small and constant positive pressure to the internal solution. Use IR-DIC with ×4 objective to identify the surface of the slice. Move the tips of electrodes to the region of interest, i.e., CA1 *stratum radiatum*. In voltage clamp mode, apply a recurrent 5 mV square voltage pulse to monitor the electrode resistance. Switch to the water immersion ×40 objective. Apply a drop of bath solution to the objective that runs down and forms a fluid bridge between the front lens and the slice. Advance electrodes into the slice ~ 50 μm beneath the surface.

**6 |** Select a viable glial cell based on an irregular soma shape with a diameter around 10 μm. Typically, one or more primary processes stemming from the soma are visible (Figure 1a). To confirm an astrocytic identify, SR101 staining can be used. However, be aware that an adverse effect on neuronal excitability has been reported with SR101 staining [17]. After establishing the whole-cell configuration, mature astrocytes can be unequivocally identified by the expression of passive conductance (Figure 1b). Alternatively, astrocytes can be identified solely and easily by SR101 staining. Switch to IR-DIC visualization.

**7 |** Approach the cell slowly with one electrode at a time. Once on-cell, see dimpling and release the electrode pressure. Apply negative potential (ranging from -70 to -80 mV) through the commander panel to facilitate seal formation. This manipulation typically results in the rapid formation of a GΩ seal. Repeat this procedure with the second electrode. Check and keep the position of first electrode relative to the cell. Finally, ensure both electrodes to form GΩ seal on the same cell.

▲CRITICAL This is the most critical step requiring careful coordination of two electrodes and readjustment of the relative position between cell soma and electrodes. Often, when the targeted cell is approached by the second electrode, the cell can move away from the first electrode. Adjust the first electrode to follow the cell when it is necessary.

? TROUBLESHOOTING(1)

**8 |** Rupture the patch of membrane isolated inside the electrode tip by negative pressure pulses to establish the whole-cell configurations. No obvious change in holding current shift associated with a decrease of seal test resistance to < 30 MΩ is an indication of successful formation of whole-cell configuration. Repeat this for the second electrode. After both whole-cell configurations are established, wait at least 5 min to allow ion equilibration between electrode solution and intracellular cytoplasm

? TROUBLESHOOTING(2)

**9 |** Set astrocyte in desired mode, e.g., with one electrode in current clamp and another electrode in voltage mode under the control of Clampex 9.2. Program both channels in either voltage or current clamp mode depending on experimental purpose.

(a) To evaluate the voltage dependence of channel activation, apply a series of command voltage steps, e.g., -210 mV to +30 mV in 40 mV increments, from the holding potential of -80 mV

(b) To monitor change of *R*_M_ (or *R*_t_) in response to drugs, e.g., GABA, apply a single negative command voltage step, e.g., -60 mV from holding potential for 20 ms.

? TROUBLESHOOTING(3)

**10 |** Analyze dual patch results.

(a) *I*-*V*_M_ relationship can be derived from the stepwise command voltages as shown in Figure 2a. Plot *I* readings from E1 against their corresponding *V*_M_ readings from electrode 2. Determine the reversal potential of the current by extrapolating the data point on the *I*-*V*_M_ curve.

(b) Plot the conductance-voltage (*G*-*V*_M_) curve by dividing the current amplitude change (ΔI) by the measured voltage step (Δ*V*_M_) at each potential. As shown in Figure 4f.

? TROUBLESHOOTING

**Step 7**, when GΩ is difficult to establish, check the following possibilities:

a) Brain slice quality is always the first thing to be considered. Ensure the preparation procedure was followed strictly; otherwise a new preparation is needed.

b) The difficulty in achieving GΩ seal increases with animal age, start your practice with P10 mice is recommended to familiarize the procedure. Young adult mice from P21 - P30 can be used in most of experiments, unless the study targets on an age related subject.

c) Make sure to apply and maintain a small positive pressure before the electrodes touching to the bath solution in step 5.

d) Apply the suction as gentle as possible and increase it in a stepwise manner until the membrane is ruptured.

e) Ensure electrode solution is freshly prepared with correct pH and osmolality.

If the seal in the first electrode is lost when the second electrode approaches the cell, pay attentions to the following:

a) Focus on the cell when the second electrode approaches the cell. Move the first electrode to follow the cell if the cell moves.

b) Adjust slice anchor and hold more tightly if too loose.

c) The deeper the targeted cell beneath the surface of slice, the more likely to encounter a cell movement during the handling the second electrode to approach the cell. Select a relatively shallow cell, ~50 μm is recommended.

d) Approach the cell gently. Avoid larger movements of the second electrode, including approaching, going up or down. If the tip of the second electrode is too shallower or deeper than cell body, withdraw the second electrode gently, adjust the position on the top of slice and re-approach.

**Step 8**, apply suction pulses either by mouth or use of a syringe. Try following options:

a) In our experience, a relatively strong and fast pulse works well to rupture membrane and achieve a relatively stable *R*_*a*_.

b) Keep the same or increase the suction pressures in the repetitive rupture attempt, release pressure immediately if membrane resistance in seal test drops suddenly.

c) Keep a small pressure and click “Zap”, and release the pressure after Zap. Repeat Zap if necessary.

d) If the seal resistance is higher than 30 MΩ, indication of *R*_t_ >30 MΩ, repeat above steps may decrease the *R*_t_ of whole-cell recording.

**Step 9**, *R*_a_ tends to increase during recording (Figure 2f), do the following to minimize it:

a) Low resistance electrodes, i.e., 2.0-2.5 MΩ, has been demonstrated to be feasible and is recommended [18]; as such, the *R*_t_ can be physically reduced to the lowest workable range.

b) Empirically, a relatively stronger suction pressure for whole-cell break-through results in a high percentage of low and stable *R*_a_ whole-cell recordings.

c) To ensure that the brain slice is anchored securely in the chamber; this prevents potential slice movement resulting from perfusion pulsatile.

d) In general, the older the animal the more difficulty to achieve a long-lasting stable *R*_a_, use young adult animals unless the experiment examining an ageing related question.

e) Use mechanically stable micromanipulators.

f) We routinely discard recordings with high *R*_a_ (or *R*_t_) from data analysis.

●**Timing**

Step 1, slice preparation: 30 min

Step 2–3, slice recovery and SR101 staining: at least 1 h

Step 4, transfer slice to recording chamber and re-equilibration: 20 min

Step 5, loading both electrodes: 10 min

Step 6, select astrocyte: 5 min

Step 7, approach the cell and form GΩ seals: 5–30 min

Step 8, break through to form dual patch recording: 3–10 min

Step 9, recording: 30 min - 4 h, depending on the experimental design

Step 10, Analysis: variable, depending on required data.

## Anticipated results

Dual patch recording was once considered to be a time-consuming technique which required considerable skill. Facilitated by the use of advanced equipment, specifically PatchStar micromanipulators and software (Scientifica, UK), a Polychrome V imaging system (Till Photonics, Germany), and a MultiClamp 700A/700B amplifier (Molecular Devices, Sunnyvale, CA), this recording mode can be performed routinely, studying 3–8 cells in a 6-hour experimental day. For experienced researcher, the dual patch single astrocyte recording can be achieved within 10 min after the cell being visual identified. Should all the steps be followed carefully, successful dual patch single astrocyte recording can be readily achieved. In some of the experiments, successful dual patch recordings recording had lasted up to 4 hours in our designed experiment. Thus, dual patch is an ideal technique for the future study of functional channels, receptors, and electrogenic transporters in astrocytes.

## Competing interests

The authors declare that they have no competing interests.

## Authors’ contributions

BM and MZ conceived the study. All the authors were involved in the design of the experiments and discussion of the results. BM and GX performed experiments and analyzed the data. BM, GX, JJE, and MZ wrote the manuscript. MZ supervised the research. All authors read and approved the final manuscript.
